# Effects on Metabolic Health after a 1-Year-Lifestyle Intervention in Overweight and Obese Children: A Randomized Controlled Trial

**DOI:** 10.1155/2012/913965

**Published:** 2011-09-19

**Authors:** Maria Waling, Catharina Bäcklund, Torbjörn Lind, Christel Larsson

**Affiliations:** ^1^Department of Food and Nutrition, Umeå University, 901 87 Umeå, Sweden; ^2^Department of Clinical Sciences, Pediatrics, Umeå University, 901 87 Umeå, Sweden

## Abstract

*Objective.* To evaluate the effect of a family-based intervention on anthropometric and metabolic markers in overweight and obese children. *Methods.* Overweight or obese 8–12 years olds (*n* = 93) were randomized into intervention or control groups. The intervention group participated in a program aiming for lifestyle changes regarding food habits and physical activity. Anthropometric measures and venous blood samples were collected from all children at baseline and after 1 year. *Results.* BMI z-scores decreased in both groups, 0.22 (*P* = 0.002) and 0.23 (*P* = 0.003) in intervention and control group, respectively, during the 1-year study, but there was no difference in BMI between the groups at 1-year measurement (*P* = 0.338). After 1 year, there was a significant difference in waist circumference, waist/hip ratio, and apolipoprotein B/A1 ratio between intervention and control group. *Conclusions.* The intervention had limited effects on anthropometrics and metabolic markers, which emphasizes the need of preventing childhood overweight and obesity.

## 1. Introduction

Along with the obesity epidemic, the prevalence of metabolic syndrome (MetS) has increased among children and adolescents [1, 2]. A recent review concluded that the worldwide prevalence of MetS among children and adolescents varies between 1.2 and 22.6% [2]. The highest rates of MetS among overweight and obese children were observed in Turkey and the US (around 40%) and the lowest rates in China, France and Italy (around 11.5%). In Europe, the prevalence of MetS vary from 0.2% among 10 year olds in Estonia, Portugal, and Denmark [3] to 21% among 4–16 year olds in Germany [4]. Apart from differences in obesity prevalence, another explanation to the great variation is the many definitions of MetS for children and adolescents [5]. 

The International Diabetes Federation recently defined MetS in children and adolescents as abdominal obesity and the presence of 2 or more other clinical features (i.e., elevated triglycerides, low HDL-cholesterol, high blood pressure, or increased plasma glucose) [6]. The most important risk factor for developing MetS is obesity [2] and the risk increases with increased levels of obesity [7]. Unhealthy dietary habits and low levels of physical activity are other important risk factors [1, 3]. Overweight and obesity as well as MetS in childhood and adolescence predict future mortality [8] and diseases such as type 2 diabetes and atherosclerotic cardiovascular disease [1, 7, 9]. Since obese children are at high risk of becoming obese adults and components of MetS track into adulthood early intervention is of great importance [1, 10, 11].

There is some evidence that early interventions aiming at managing obesity could reduce the risk of developing MetS and improve metabolic health [12]. The initial treatment of MetS in obese children and adolescents is the reduction of body mass index (BMI) [10], that is, the same as for managing childhood overweight and obesity [13]. A recent Cochrane review stated that family-based, lifestyle interventions with behavioural programs aiming at improving physical activity and food habits appear to be the most effective treatment for childhood overweight and obesity [14]. However, many studies aiming at preventing further development of overweight and obesity have limited results [15, 16] and there is a need of studies trying new methods, for example, the use of internet as a way of communicating with children and parents [17]. 

The aim of the present study was to evaluate the effect of a family-based intervention program on anthropometric and metabolic markers in 8–12-year-old overweight and obese children.

## 2. Methods

### 2.1. Study Design

This study was a parallel group, randomized controlled trial where children were randomized into either an intervention or control group. Neither the researchers nor the participants were blinded. However, the research nurses performing anthropometric and biochemical measurements were not informed about the children's group allocation. The anthropometric and biochemical measurements were done at the Pediatric Clinical Research Unit, Department of Pediatrics, Umeå University, and the intervention sessions were conducted at the Department of Food and Nutrition, Umeå University. Children allocated to the control group participated in one information session. Apart from that, they only participated in the same measurements as the intervention group.

### 2.2. Participants

**Figure 1:**
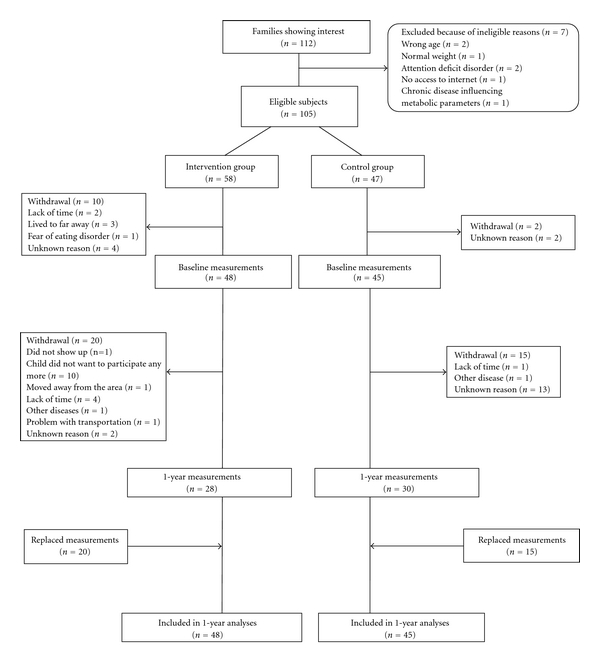
Flowchart of participants in a 1 year randomized controlled intervention study with overweight and obese children.

All families, regardless if they were normal weight or overweight, with children born between 1995 and 1998 living in or near the northern Swedish city Umeå (*n *= 6290) were informed about the study by a letter from the researchers. To participate in the study, the children had to have an age and gender adjusted BMI ≥ 25 kg/m^2^ [18], be born 1995–1998, and live in or nearby the city of Umeå. Excluded were children who had chronic diseases that could influence metabolic parameters, attention deficit disorders, or lack of access to internet. To obtain a study power of 80% with *α* = 0.05 and to detect a difference in BMI of 1.6 kg/m^2^ between intervention and control children, 84 participants were needed. However, to allow for a dropout, we aimed to recruit 120 children. Recruitment and randomization occurred at four different time points, October 2006 and in January, March, and May 2007. In total, 112 families showed interest to participate and were contacted by phone to ascertain eligibility. Seven children did not meet inclusion criteria and were excluded. As a result, 105 children (53 girls and 52 boys) were included in the study (Figure 1). The children were consecutively randomised (1 : 1) and stratified by gender into either an intervention group or a control group by the researchers. After inclusion, 12 of the 105 randomized children changed their mind regarding participation (before having done baseline measurements) which resulted in 93 children who participated in baseline measurements (Figure 1). During the time period from inclusion to the baseline measurements, ranging from 3 days to 2 months, the BMI classification of 3 children, 2 allocated to the intervention group and 1 to the control group, changed from overweight to normal weight. These 3 children remained in the group, they were randomized to and continued to participate in the study. Of the recruited children, 10–15 were allocated to each of 4 subintervention groups and 4 subcontrol groups, starting on 4 different occasions in time. Eleven more children were randomized to the last intervention group than to the last control group, to compensate for dropouts that had occurred in previously three intervention groups. 

Written informed consent was obtained from the children's parent and verbal consent was ascertained from each child through their parents. The study was approved by the Regional Research Ethics Review Board (ref number: 05–088M) and all applicable institutional and governmental regulations concerning the ethical use of human volunteers were followed during the study.

### 2.3. Intervention

The family-based intervention was based on principles of behavioural [19, 20] and solution-oriented group work [21], where both children and parents participated. Focus was on improving present behaviours related to food and physical activities in a group setting. The families had their own goals that they worked toward. The aim of the intervention was to improve food and physical activity habits, and the intervention has been described in more details previously [22, 23]. In summary, the first year of the intervention program consisted of 14 sessions 1-2 times per month. Five sessions concerned food habits, 4 physical activities, and the remaining 5 sessions focused on behavioural change as well as working towards personal goals and motivation. Between sessions, participants were given home assignments that were related to the theme of the upcoming session and contact was held through a web platform where the participants could report home assignments or communicate with other participants or leaders between the sessions. The recommended dietary intake and physical activity level were in line with national recommendations given to Swedish children [24]. The mean attendance rate at the 5 sessions regarding food among intervention children who participated during the first year (a mean of 7 children participated per group) was 3.4 (range 1–5) children per session. The corresponding rate for the 4 sessions regarding physical activity in groups was 4.1 (range 2–7) children per session and for the remaining 5 sessions regarding behavioural change and self-esteem the attendance rate was 5.3 (range 3–8) children per session. All group sessions were led by the authors M. Waling (with a food and nutrition degree) and C. Bäcklund (registered physiotherapist), and at some of the sessions other health professionals were invited to colead the sessions. At 3 sessions, a registered dietician was invited, at 1 session a paediatric physician was invited, and at 3 sessions a child psychologist was invited. All sessions were planned before the intervention started and each session had a manual which was followed by the leaders. 

### 2.4. Outcome Measures

Outcome measures were obtained at baseline and 1 year into the study in both intervention and control groups. Height and weight were measured with the children lightly clothed and without shoes. Height was measured to the nearest 0.1 cm with a wall stadiometer (Hyssna Measuring Equipment AB, Sweden), and weight was measured to the nearest 0.1 kg with an electronic scale (AJ Medical, Sweden). The main outcome of the study, BMI, was calculated as weight (kg)/height (m)^2^ and converted to BMI *z*-scores by using both US reference data [25] and a Swedish reference dataset [26]. Children were classified as normal weight, overweight, or obese using the International Obesity Task Force (IOTF) definitions [18], and parents' weight status was classified using the World Health Organization's definitions [27]. Waist circumference measurements were recorded to the nearest 0.1 cm midway between the tenth rib and the iliac crest with children in a standing position using a nonelastic flexible tape. Sagittal abdominal diameter was measured to the nearest 0.1 cm using a ruler with the child in a supine position from the bed to the top of the abdomen. Puberty stage was estimated according to Tanner [28, 29] and puberty was categorized into 2 groups: pre-pubertal (Tanner stage 1) and pubertal (Tanner stage 2–4), no child had reached Tanner stage 5. Body composition analysis was performed using dual energy X-ray absorptiometry (DEXA) (Lunar Prodigy whole-body scanner GE Medical Systems, Madison,Wis, USA), with the child in a supine position. Body fat content is expressed as absolute values (kilograms) and as percent fat (fat mass %), and truncal fat expressed as percent fat (truncal fat mass %) in the soft tissue of the trunk. Fat mass index (FMI, kg/m^2^) was calculated as fat mass (kg)/height (m)^2^ [30]. 

Blood pressure was measured on the right arm using an electronic blood pressure device (Welch Allyn Spot Vital Signs, Welch Allyn AB, Sweden) after supine rest for 5 minutes. Venous blood samples were collected after overnight fasting and analysed for plasma glucose, serum lipids [total cholesterol (TC), high-density lipoprotein cholesterol (HDL-C), low-density lipoprotein cholesterol (LDL-C), triglycerides (TG), apo lipoprotein A_1_ (apo A_1_) and apo lipoprotein B (apo B)], insulin, and HbA1c. Homeostatic model assessment index (HOMA-index) was calculated as (insulin × glucose/22.5) [31]. The children were classified as having MetS using the definitions of the International Diabetes Federation [6]; waist circumference ≥ 90th percentile and the presence of 2 or more other clinical features (i.e., elevated TC, low HDL-C, high blood pressure, or increased glucose). Reference data collected in a cohort of 10-year-old children from Umeå was used when defining the 90th percentile of waist circumference [32].

### 2.5. Statistical Analysis

Statistical analyses were performed using SPSS version 17.0 (SPSS INC., Chicago, Ill, USA) and the significance level was set at *P* < 0.05. Results are expressed as means and standard deviation or proportions. Independent *t*-test and one-way analysis of covariance (ANCOVA) with adjustment for baseline data were used to analyze differences between intervention and control groups. Paired samples *t*-test were performed to determine differences within intervention and control groups, respectively, between baseline and 1-year measurement. Differences in proportions were tested with the chi-square test. Relationship between BMI and truncal fat was investigated using Pearson product-moment correlation coefficient.

Results were analysed both per protocol and on an intention-to-treat analysis basis, that is, all children were analyzed in the group they originally were randomized to. In the intention-to treat-analysis the principle of carrying the last observation (in this study baseline measurements were used) forward was used in children who dropped out before the 1 year measurement.

## 3. Results

From baseline to the 1-year measurement, 42% of the children in the intervention group and 33% of the children in the control group dropped out (Figure 1), which left 58 children who had completed the 1-year measurement. The proportion of dropouts did not differ between the groups (*P* = 0.407). In the intention-to-treat analysis where the principle of the last value carry forward was used, the 35 children who had dropped used baseline measurements as a proxy for the 1-year measurement. 

Forty-eight children (44% girls) in the intervention group completed baseline measurement of which 38% were classified as obese and 45% had at least one parent classified as being obese. In the control group, 45 children (58% girls) completed the baseline measurements, 24% were classified as being obese and 36% had at least one parent classified as being obese. No differences were seen between the groups regarding gender distribution (*P* = 0.176), prevalence of overweight and obesity (*P* = 0.109), or prevalence of having at least one parent classified as being obese (*P* = 0.372). The only difference noted at baseline was a 2.2% higher fat mass in the intervention group compared to the control group (Table 1).

After 1-year participation, there was no difference in BMI, BMI *z*-score, or proportions of children classified as being normal weight, overweight, or obese between the intervention and control group (Table 2). However, in contrast to the intervention group, the proportion of children changing weight status within the control group was statistically significant (*P* < 0.001) after 1-year participation, with 2 less children classified as obese and 6 more children being classified as normal weight. In the intervention group, 4 less children were classified as obese and 2 more children were classified as normal weight after 1-year participation but the change was not statistically significant (*P* = 0.182). There were children in both groups who decreased BMI during the study period without a change in classification (e.g., if classified as overweight at baseline still classified as overweight at 1-year measurement); in the intervention group, there were 4 children with a BMI decrease ranging from 0.28–2.20 kg/m^2^, and in the control group there were 2 children with a BMI decrease of 0.41–0.72 kg/m^2^.

Both the intervention and control group decreased BMI *z*-scores from baseline to 1-year measurement, regardless of reference population (Swedish or the US) (Table 3). There was no statistically significant change in the proportion of children having a BMI *z*-score above +2 SD during first year of the study within the intervention or control group (*P* = 0.141 or *P* = 0.258, resp.) independent of reference population.

After 1-year of intervention, there was a statistically significant difference between intervention and control group in waist circumference, waist/hip ratio, and ApoB/A_1_ when baseline measurements had been carried forward (Table 3). However, these differences were not visible in the per protocol analysis. The waist/hip ratio did not change significantly in the intervention group but it increased by 0.02 (*P* = 0.042) in the control group when analysing according to the last observation carried forward and similar results were seen when analysing per protocol. The apoB/A_1_ quotient did not change significantly in the intervention group but it increased by 0.03 (*P* = 0.032) in the control group when using the last observation carried forward and similar results was seen when analysing per protocol, resulting in a significant difference between the two groups after 1 year of participation (*P* = 0.041).

The relationship between BMI and truncal fat (expressed as %truncal fat mass) was strong at baseline in both the intervention group, *r* = 0.75 (*P* ≤ 0.001), and the control group, *r* = 0.62 (*P* ≤ 0.001). After 1 year, when analysing, according to the last observation carried forward the relationship was 0.76 (*P* ≤ 0.001) in the intervention group and 0.73 (*P* ≤ 0.001) in the control group. The relationships were not changed when analysing per protocol.

At baseline, 3 children in the study were defined as having MetS, 1 participant in the intervention group, and 2 participants in the control group. At 1 year measurement 3 children in the intervention group and 2 in the control group fulfilled the MetS definition. The nonstatistically significant increase in MetS prevalence in the intervention group was explained by increased TG levels in 2 children.

## 4. Discussion

After 1-year participation in the present study, BMI *z*-scores decreased in both groups and there were no differences in BMI or BMI *z*-score between the groups. The family-based intervention program designed to improve health of overweight and obese children through lifestyle changes resulted in limited effects on anthropometric and metabolic measurements. 

In accordance with the present study, many similar intervention studies with the same aim as ours show no difference in BMI between intervention and control group 1.5–2 years into the intervention. One study performed in Finland that included 7–9-year-old obese children in a family group treatment showed no intervention effect on BMI 2 and 3 years after baseline [15]. Further, Williamson et al. showed no intervention effect on BMI after a 2-year web-based intervention on 11–15-year-old overweight and obese girls [17]. Similar results with no intervention effect on BMI were seen among 8–12-year-old extremely obese children at 1.5 year follow-up [16]. On the other hand, one study performed in the US on 8–16-year-old obese children did show a 2.8 kg/m^2^ lower BMI in the intervention group compared to control group at a 2-year followup [33].

Even though the intervention did not succeed in decreasing the intervention groups' BMI in relation to the control group after 1 year of participation, the stagnated BMI (which naturally increases as children grow) and the decreased BMI *z*-score [26] in both groups indicate that both groups have been affected. One reason for this could be that merely the participation in a study with focus on a healthy lifestyle could be an incitement for lifestyle change [34]. Another reason could be the focus on the increased prevalence of overweight in media the past years. The possible effect of society on BMI and BMI *z*-score in the present study is supported by studies that show stagnation and in some cases a decrease in prevalence of childhood overweight and obesity in Sweden [29–31]. 

In the present study, the prevalence of MetS (3% at baseline and 5% at 1-year measurement) was low compared to the reported prevalence of 11–32%, among overweight and obese children in Europe [2]. Despite the decreased BMI *z*-score, we saw no effect on biochemical parameters or blood pressure. This may be explained by the fact that a majority of the children were within the normal range at baseline and consequently major improvements could not be expected. Some studies suggest that a reduction of ≥0.25 in BMI *z*-score is needed for a minor improvement in metabolic markers among obese adolescent [35] and that a decrease of ≥0.5 units is needed for a major improvement [4, 36]. It is possible that we would have seen different results if we had included children that had abnormal metabolic values to begin with. 

In the present study, we had mainly families representing those living in the town of Umeå, a town with a high educational level. It is most likely that families that agree to participate in a study like the present one are more motivated than those who decline participation. These characteristics of the study population are a limiting factor when it comes to the generalization of the results.

A strength of the present study is the randomized controlled trial design but the sample size is small and the study had a high dropout rate. Even though several recruitment efforts were made, including expanding the inclusion criteria to a larger age and BMI span, expanding the geographical catchment area and even though all families in the area with children in the specific age group were sent an invitation, we were not able to recruit a larger number of participants. We also had a higher dropout than expected even though high dropout rates are quite common in intervention studies aiming at treating childhood overweight and obesity and may vary between 12 and 52% [14]. The most common reason of intervention families for leaving the present study was that the child was not interested in participating, while most of the control families left the study without explanation. The large dropout rate and consequently low study power makes it difficult to detect small differences between intervention and control group as well as to generalize the results. This is a major limitation of the study and to increase the study power a last observation carried forward strategy was used to replace dropouts. The last observation carried forward principle is one of the most commonly used strategies to deal with drop outs [37], even though it has some disadvantages [38]. A disadvantage to be considered is dilution of the intervention effect, and in the present study 42% and 33% of the baseline data of the intervention, respectively, control group was used as proxy for the 1-year measurements. To control for this all analyses were also made per protocol showing similar results, however then there might not be enough power in the study to be able to detect a small intervention effect [38]. Another limitation of the present study is the relatively many hypotheses that were tested which increases the risk of chance findings. 

In conclusion, the family-based lifestyle programme had limited effects on anthropometric and metabolic outcomes of the overweight and obese children. This strongly supports the idea that efforts should primarily be aimed at primary prevention of overweight and obesity.

## Figures and Tables

**Table 1:** Baseline characteristics; anthropometric measures, and fasting blood values, of overweight and obese children presented as mean (standard deviation).

	Intervention group *n* = 48	Control group *n* = 45 or 44*	P**
Age (years)	10.5 (1.15)	10.5 (1.02)	0.914
Weight (kg)	52.1 (9.95)	50.4 (9.99)	0.402
Height (cm)	149 (7.86)	149 (8.64)	0.951
BMI (kg/m^2^)	23.4 (2.79)	22.6 (2.39)	0.148
BMI *z*-score***	2.03 (0.88)	1.77 (0.71)	0.130
BMI *z*-score^†^	3.23 (1.34)	2.75 (1.04)	0.057
Waist circumference (cm)	78.0 (10.1)	74.7 (8.80)	0.094
Hip circumference (cm)	89.4 (7.53)	87.1 (7.57)	0.145
Waist/hip ratio	0.87 (0.07)	0.86 (0.07)	0.352
Sagittal abdominal diameter (cm)	17.6 (2.53)	17.0 (1.82)	0.153
Fat mass (kg)	19.8 (6.19)	17.9 (4.97)	0.121
Fat mass (%)	39.2 (6.17)	37.0 (4.33)	**0.044**
Truncal fat mass (%)	38.4 (7.46)	36.2 (5.42)	0.115
Diastolic blood pressure (mm Hg)	66.6 (5.60)	66.7 (6.14)	0.959
Systolic blood pressure (mm Hg)	112 (8.92)	113 (8.80)	0.739
Total cholesterol (mmol/L)	4.44 (0.91)	4.51 (0.82)	0.711
LDL-cholesterol (mmol/L)	2.58 (0.78)	2.71 (0.74)	0.415
HDL-cholesterol (mmol/L)	1.46 (0.32)	1.35 (0.28)	0.104
Triglycerides (mmol/L)	0.88 (0.53)	0.95 (0.39)	0.498
Apolipoprotein B (mg/L)	745 (173)	782 (174)	0.316
Apolipoprotein A_1 _(mg/L)	1350 (182)	1330 (169)	0.708
Apolipoprotein B/A_1_ ratio	0.56 (0.14)	0.59 (0.14)	0.280
Glucose (mmol/L)	4.52 (0.43)	4.65 (0.42)	0.157
Insulin (mU/L)	10.3 (6.77)	10.2 (6.82)	0.976
HOMA-index	2.06 (1.37)	2.18 (1.65)	0.722
Hb A_1_c (%)	4.03 (0.24)	3.98 (0.27)	0.337

*One child refused to give a blood sample.

**P value for difference between groups from independent samples *t*-test.

***Reference population from CDC in the USA [21].

^†^Reference population from a Swedish population-based study [22].

**Table 2:** Proportion of normal weight, overweight and obesity among overweight, and obese children, presented as number (%).

	Baseline			1 year		
	Intervention *n* = 48	Control *n* = 45	P*	Intervention *n* = 48	Control *n* = 45	P*
BMI**						
Normal weight	2 (4)	1 (2)		4 (8)	7 (16)	
Overweight	28 (58)	33 (73)	0.311	30 (63)	29 (64)	0.431
Obese	18 (38)	11 (24)		14 (29)	9 (20)	

*Difference in proportions between intervention group and control group analyzed with Chi-square test.

**Classification according to IOTF [16].

**Table 3:** Anthropometric measures and fasting blood values after a 1-year intervention in overweight and obese children presented as mean (standard deviation).

	Intervention group						Control group							
	Last observation carried forward			Per protocol			Last observation carried forward			Per protocol				
	(*n* = 48)			(*n* = 28)			(*n* = 45 or 44)*			(*n* = 30)				
	1 year	Change from baseline	P**	1 year	Change from baseline	P**	1 year	Change from baseline	P**	1 year	Change from baseline	P**	P***	*P* ^†^
Weight (kg)	54.9 (10.4)	2.78 (0.50)	**<0.001**	56.5 (11.4)	4.76 (3.29)	**<0.001**	54.0 (11.7)	3.65 (4.17)	**<0.001**	55.4 (11.5)	5.47 (4.01)	**<0.001**	0.509	0.207
Height (cm)	152 (8.38)	3.67 (3.36)	**<0.001**	154 (9.55)	6.29 (1.59)	**<0.001**	153 (9.33)	4.48 (3.53)	**<0.001**	155 (8.33)	6.71 (1.82)	**<0.001**	0.324	0.402
BMI (kg/m^2^)	23.5 (2.70)	0.08 (0.95)	0.572	23.7 (2.74)	0.13 (0.24)	0.576	22.8 (2.86)	0.20 (1.12)	0.240	22.9 (3.00)	0.30 (1.37)	0.242	0.747	0.338
BMI *z*-score^††^	1.81 (0.82)	−0.22 (0.37)	**<0.001**	1.71 (0.81)	−0.37 (0.08)	**<0.001**	1.55 (0.82)	−0.23 (0.48)	**0.003**	1.52 (0.90)	−0.34 (0.56)	**0.003**	0.844	0.523
BMI *z*-score^†††^	2.94 (1.22)	−0.29 (0.62)	**0.002**	2.79 (1.19)	−0.50 (0.74)	**0.002**	2.57 (1.21)	−0.18 (0.59)	0.050	2.56 (1.32)	−0.27 (0.71)	0.050	0.536	0.432
Fat mass index (kg fat/m^2^)	13.4 (3.68)	0.25 (1.25)	0.171	13.7 (3.86)	0.47 (1.65)	0.154	12.5 (3.65)	0.55 (1.60)	**0.029**	12.8 (4.06)	0.84 (1.93)	**0.028**	0.318	0.432
Waist circumference (cm)	79.3 (9.99)	1.29 (3.97)	**0.029**	80.2 (9.75)	2.21 (5.04)	**0.028**	78.1 (9.14)	3.49 (6.44)	**0.001**	79.8 (9.34)	5.23 (7.31)	**<0.001**	**0.043**	0.211
Hip circumference (cm)	90.4 (7.65)	1.07 (2.82)	**0.011**	91.0 (7.50)	1.84 (3.52)	**0.010**	89.0 (8.29)	1.96 (3.98)	**0.002**	90.0 (8.03)	2.94 (4.58)	**0.001**	0.932	0.119
Waist/hip ratio	0.88 (0.07)	0.00 (0.03)	0.393	0.88 (0.06)	0.01 (0.04)	0.397	0.88 (0.06)	0.02 (0.06)	**0.042**	0.89 (0.06)	0.03 (0.08)	**0.041**	**0.029**	0.449
Sagittal abdominal diameter (cm)	17.9 (2.22)	0.28 (1.14)	0.097	18.2 (2.09)	0.48 (1.48)	0.098	17.5 (2.29)	0.54 (1.28)	**0.007**	17.9 (2.26)	0.82 (1.50)	**0.006**	0.590	0.387
Fat mass (kg)	20.6 (6.31)	0.88 (2.07)	**0.005**	21.3 (6.84)	2.92 (1.51)	**<0.001**	19.3 (6.37)	1.43 (2.66)	**0.001**	20.0 (6.94)	2.19 (3.04)	**0.001**	0.483	0.329
Fat mass (%)	39.0 (6.11)	−0.21 (2.26)	0.514	39.0 (5.55)	−0.39 (3.06)	0.519	36.9 (5.82)	−0.16 (3.34)	0.761	36.8 (6.67)	−0.24 (4.16)	0.763	0.965	0.957
Truncal fat mass (%)	38.0 (7.47)	−0.38 (2.63)	0.312	38.2 (7.22)	−0.70 (3.54)	0.316	35.9 (7.46)	−0.29 (3.98)	0.640	35.8 (8.48)	−0.44 (4.96)	0.643	0.830	0.780
Diastolic blood pressure (mm Hg)	66.7 (5.44)	0.10 (3.49)	0.837	67.8 (5.06)	0.18 (0.87)	0.839	66.6 (6.00)	−0.07 (5.07)	0.929	67.5 (5.74)	−0.10 (6.28)	0.930	0.272	0.789
Systolic blood pressure (mm Hg)	111 (8.58)	−0.58 (5.81)	0.490	112 (8.89)	−1.00 (7.64)	0.494	114 (9.28)	1.38 (6.99)	0.193	115 (8.55)	2.07 (8.52)	0.194	0.758	0.218
Total cholesterol (mmol/L)	4.32 (0.91)	−0.12 (0.49)	0.087	4.10 (0.76)	−0.21 (0.63)	0.087	4.33 (0.82)	−0.18 (0.37)	**0.002**	4.27 (0.73)	−0.27 (0.42)	**0.002**	0.520	0.729
LDL-cholesterol (mmol/L)	2.54 (0.78)	−0.05 (0.33)	0.342	2.39 (0.62)	−0.79 (0.43)	0.346	2.64 (0.75)	−0.08 (0.35)	0.159	2.57 (0.76)	−0.11 (0.42)	0.160	0.828	0.704
HDL-cholesterol (mmol/L)	1.39 (0.35)	−0.07 (0.18)	**0.011**	1.32 (0.32)	−0.12 (0.22)	**0.009**	1.28 (0.29)	−0.07 (0.15)	**0.005**	1.30 (0.30)	−0.10 (0.18)	**0.004**	0.983	0.513
LDL/HDL ratio	1.91 (0.60)	0.06 (0.27)	0.115	1.89 (0.52)	0.11 (0.35)	0.115	2.16 (0.74)	0.10 (0.42)	0.136	2.13 (0.87)	0.14 (0.51)	0.137	0.711	0.846
Triglycerides (mmol/L)	0.87 (0.63)	−0.01 (0.43)	0.825	0.86 (0.59)	−0.02 (0.56)	0.827	0.89 (0.40)	−0.06 (0.44)	0.338	0.88 (0.43)	−0.10 (0.54)	0.341	0.286	0.732
Apolipoprotein B (g/L)	0.73 (0.17)	−0.01 (0.07)	0.243	0.70 (0.13)	−20 (0.08)	0.246	0.76 (0.16)	−0.03 (0.09)	**0.048**	0.79 (0.16)	−0.04 (0.10)	**0.048**	0.860	0.907
Apolipoprotein A_1 _(g/L)	1.29 (0.20)	−0.05 (0.13)	**0.009**	1.25 (0.18)	−0.09 (0.16)	**0.008**	1.23 (0.15)	−0.10 (0.15)	**<0.001**	1.22 (0.14)	−0.16 (0.16)	**<0.001**	0.060	0.411
Apolipoprotein B/A_1_ ratio	0.57 (0.13)	0.01 (0.05)	0.134	0.56 (0.11)	0.02 (0.07)	0.135	0.62 (0.13)	0.03 (0.08)	**0.032**	0.61 (0.15)	0.04 (0.02)	**0.031**	**0.041**	0.342
Glucose (mmol/L)	4.58 (0.42)	0.06 (0.25)	0.132	4.62 (0.36)	0.09 (0.33)	0.133	4.62 (0.41)	−0.02 (0.27)	0.623	4.64 (0.38)	−0.31 (0.34)	0.626	0.565	0.494
Insulin (mU/L)	11.2 (5.76)	0.90 (4.09)	0.136	12.1 (4.90)	1.54 (5.30)	0.137	10.4 (5.71)	0.21 (5.37)	0.797	10.7 (5.62)	0.32 (6.64)	0.799	0.699	0.660
HOMA-index	2.28 (1.19)	0.21 (0.93)	0.122	2.49 (1.08)	0.36 (1.21)	0.123	2.20 (1.34)	0.02 (1.26)	0.896	2.26 (1.33)	0.04 (1.56)	0.897	0.827	0.716
HbA_1_c (%)	4.11 (0.23)	0.07 (0.15)	**0.002**	4.15 (0.24)	0.13 (0.19)	**0.001**	4.03 (0.27)	0.04 (0.13)	**0.037**	4.08 (0.26)	0.07 (0.16)	**0.036**	0.612	0.127

*One child refused to give blood sample.

**P values for difference between baseline and 1-year analyzed by paired samples *t*-test.

***P values for difference between groups at 1-year analyzed by the last observation carried forward principle with a one-way analysis of covariance.

^†^ *P* values for difference between groups at 1-year analyzed per protocol with a one-way analysis of covariance.

^††^Reference population from the US [21].

^†††^Reference population from Sweden [22].
